# Supplementary material for: From sequence to enzyme mechanism using multi-label machine learning
Source: BMC Bioinformatics. 2014 May 19;15:150. doi: 10.1186/1471-2105-15-150 (PMC4229970; doi:10.1186/1471-2105-15-150)
Supplement: Additional file 2 — Java code of ml2db. Additional file ml2db_code.tar.gz contains the Java source code to run the multi-label machine learning experiments and save the results to database. The code’s Javadoc is included. [file 1471-2105-15-150-S2.zip › additional file 2/ml2db/ml2db/doc/main/ml2db/al/learning/ExperimentDetails.html]

ExperimentDetails


JavaScript is disabled on your browser.


- Overview
- Package
- Class
- Use
- Tree
- Deprecated
- Index
- Help

- Prev Class
- Next Class

- Frames
- No Frames

- All Classes

- Summary:
- Nested |
- Field |
- Constr |
- Method

- Detail:
- Field |
- Constr |
- Method


main.ml2db.al.learning

## Class ExperimentDetails

- java.lang.Object
- - uk.ac.standrews.utils.main.Initialised
  - - main.ml2db.al.learning.ExperimentDetails

- All Implemented Interfaces:
  :   uk.ac.standrews.utils.main.Initialisable

  ---

    

  ```
  public class ExperimentDetails
  extends uk.ac.standrews.utils.main.Initialised
  ```

  Manages the data for an active learning experiment: experiment id, arff file
  used, algorithm for training/testing, type of distance used (Euclidean etc.),
  type of utility metric used to pick the next query instance to be labelled,
  timestamp etc. It also stores these data in database and provides the
  experiment record id to be attached to the results obtained.

  Version:
  :   8 Jul 2011

  Author:
  :   Luna De Ferrari ldf(at)st-andrews.ac.uk

- - ### Field Summary

    - ### Fields inherited from class uk.ac.standrews.utils.main.Initialised

      `m_initialisationIsCorrect`
  - ### Constructor Summary

    Constructors

    Constructor and Description

    `ExperimentDetails(IdMultiLabelInstances unlabelled,
    MulanLearner algorithm,
    java.lang.String distanceFunction,
    DbManagers dbManagers,
    UtilityMetric utilityMetric,
    java.lang.String imagesPath)`

    With set of unlabelled instances (no arff record identifier)

    `ExperimentDetails(int labelledArffId,
    int unlabelledArffId,
    MulanLearner algorithm,
    java.lang.String distanceFunction,
    DbManagers dbManagers,
    UtilityMetric utilityMetric,
    java.lang.String imagesPath)`

    Both labelled and unlabelled arff record identifier.

    `ExperimentDetails(int unlabelledArffId,
    MulanLearner algorithm,
    java.lang.String distanceFunction,
    DbManagers dbManagers,
    UtilityMetric metric,
    java.lang.String imagesPath)`

    Only Unlabelled arff record identifier.
  - ### Method Summary

    Methods

    | Modifier and Type | Method and Description |
    | --- | --- |
    | `MulanLearner` | `getAlgorithm()` |
    | `DbManagers` | `getArffAndResultsDbManagers()` |
    | `java.lang.String` | `getDistanceFunction()` |
    | `java.lang.String` | `getEvaluationResultsFile()` |
    | `int` | `getExperimentId()` |
    | `java.lang.String` | `getImagesPath()` |
    | `int` | `getLabelledArffId()` |
    | `IdMultiLabelInstances` | `getLabelledInstances()` |
    | `IdMultiLabelInstances` | `getLabInstances()` |
    | `uk.ac.standrews.utils.main.database.DbManager` | `getResultsDbManager()` |
    | `uk.ac.standrews.utils.main.database.TableRow` | `getTableRow()` |
    | `int` | `getUnlabelledArffId()` |
    | `IdMultiLabelInstances` | `getUnlabelledInstances()` |
    | `IdMultiLabelInstances` | `getUnlabInstances()` |
    | `UtilityMetric` | `getUtilityMetric()` |
    | `void` | `setLabInstances(IdMultiLabelInstances labInstances)` |
    | `void` | `setUnlabInstances(IdMultiLabelInstances unlabInstances)` |

    - ### Methods inherited from class uk.ac.standrews.utils.main.Initialised

      `initialisationIsCorrect, setInitialisation, setInitialisationIsCorrect, setInitialisationNotCorrect`
    - ### Methods inherited from class java.lang.Object

      `equals, getClass, hashCode, notify, notifyAll, toString, wait, wait, wait`

- - ### Constructor Detail


    - #### ExperimentDetails

      ```
      public ExperimentDetails(IdMultiLabelInstances unlabelled,
                       MulanLearner algorithm,
                       java.lang.String distanceFunction,
                       DbManagers dbManagers,
                       UtilityMetric utilityMetric,
                       java.lang.String imagesPath)
      ```

      With set of unlabelled instances (no arff record identifier)

      Parameters:
      :   `unlabelledArffId` -
      :   `algorithm` -
      :   `distanceFunction` -
      :   `utilityMetric` -
      :   `arffDbManager` -
      :   `resultsDbManager` -
      :   `imagesPath` - the path to save graphical representations of the active
          learning experiment
      :   `resultsDirectory` - the path to save the evaluation results to


    - #### ExperimentDetails

      ```
      public ExperimentDetails(int labelledArffId,
                       int unlabelledArffId,
                       MulanLearner algorithm,
                       java.lang.String distanceFunction,
                       DbManagers dbManagers,
                       UtilityMetric utilityMetric,
                       java.lang.String imagesPath)
      ```

      Both labelled and unlabelled arff record identifier.

      Parameters:
      :   `labelledArffId` -
      :   `unlabelledArffId` -
      :   `algorithm` -
      :   `distanceFunction` -
      :   `utilityMetric` -
      :   `arffDbManagerPropsFile` -
      :   `resultsDbManagerPropsFile` -


    - #### ExperimentDetails

      ```
      public ExperimentDetails(int unlabelledArffId,
                       MulanLearner algorithm,
                       java.lang.String distanceFunction,
                       DbManagers dbManagers,
                       UtilityMetric metric,
                       java.lang.String imagesPath)
      ```

      Only Unlabelled arff record identifier.

      Parameters:
      :   `unlabelledArffId` -
      :   `algorithm` -
      :   `distanceFunction` -
      :   `dbManagers` -
      :   `metric` -
  - ### Method Detail


    - #### getAlgorithm

      ```
      public MulanLearner getAlgorithm()
      ```


    - #### getArffAndResultsDbManagers

      ```
      public DbManagers getArffAndResultsDbManagers()
      ```


    - #### getDistanceFunction

      ```
      public java.lang.String getDistanceFunction()
      ```


    - #### getEvaluationResultsFile

      ```
      public java.lang.String getEvaluationResultsFile()
      ```

      Returns:
      :   m\_evaluationResultsFile


    - #### getExperimentId

      ```
      public int getExperimentId()
      ```


    - #### getImagesPath

      ```
      public java.lang.String getImagesPath()
      ```

      Returns:
      :   m\_imagesPath


    - #### getLabelledArffId

      ```
      public int getLabelledArffId()
      ```


    - #### getLabelledInstances

      ```
      public IdMultiLabelInstances getLabelledInstances()
      ```


    - #### getLabInstances

      ```
      public IdMultiLabelInstances getLabInstances()
      ```


    - #### getResultsDbManager

      ```
      public uk.ac.standrews.utils.main.database.DbManager getResultsDbManager()
      ```


    - #### getTableRow

      ```
      public uk.ac.standrews.utils.main.database.TableRow getTableRow()
      ```


    - #### getUnlabelledArffId

      ```
      public int getUnlabelledArffId()
      ```


    - #### getUnlabelledInstances

      ```
      public IdMultiLabelInstances getUnlabelledInstances()
      ```


    - #### getUnlabInstances

      ```
      public IdMultiLabelInstances getUnlabInstances()
      ```


    - #### getUtilityMetric

      ```
      public UtilityMetric getUtilityMetric()
      ```


    - #### setLabInstances

      ```
      public void setLabInstances(IdMultiLabelInstances labInstances)
      ```


    - #### setUnlabInstances

      ```
      public void setUnlabInstances(IdMultiLabelInstances unlabInstances)
      ```


- Overview
- Package
- Class
- Use
- Tree
- Deprecated
- Index
- Help

- Prev Class
- Next Class

- Frames
- No Frames

- All Classes

- Summary:
- Nested |
- Field |
- Constr |
- Method

- Detail:
- Field |
- Constr |
- Method
